# Biocatalysis: A smart and green tool for the preparation of chiral drugs

**DOI:** 10.1002/chir.23498

**Published:** 2022-08-05

**Authors:** Giacomo Rossino, Marina Simona Robescu, Ester Licastro, Claudia Tedesco, Ilaria Martello, Luciana Maffei, Gregory Vincenti, Teodora Bavaro, Simona Collina

**Affiliations:** ^1^ Department of Drug Sciences University of Pavia, Viale Taramelli Pavia Lombardia Italy

**Keywords:** biocatalysis, chiral resolution, enantioselective synthesis, green chemistry, homochiral drugs

## Abstract

Over the last decades, biocatalysis has achieved growing interest thanks to its potential to enable high efficiency, high yield, and eco‐friendly processes aimed at the production of pharmacologically relevant compounds. Particularly, biocatalysis proved an effective and potent tool in the preparation of chiral molecules, and the recent innovations of biotechnologies and nanotechnologies open up a new era of further developments in this field. Different strategies are now available for the synthesis of chiral drugs and their intermediates. Enzymes are green tools that offer several advantages, associated both to catalysis and environmentally friendly reactants. Specifically, the use of enzymes isolated from biological sources or of whole‐cell represents a valuable approach to obtain pharmaceutical products. The sustainability, the higher efficiency, and cost‐effectiveness of biocatalytic reactions result in improved performance and properties that can be translated from academia to industry. In this review, we focus on biocatalytic approaches for synthesizing chiral drugs or their intermediates. Aiming to unveil the potentialities of biocatalysis systems, we discuss different examples of innovative biocatalytic approaches and their applications in the pharmaceutical industry.

## Graphical abstract

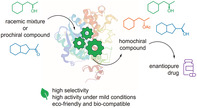

## 1 INTRODUCTION

**FIGURE 1:**
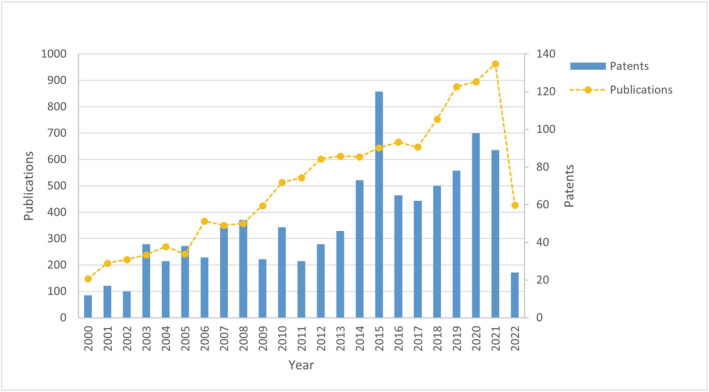
Number of documents per year on biocatalysis applications for the preparation of chiral drugs. The number of publications on scientific journals is reported as a yellow dotted line; the number of patents is reported as blue bars (source: Scopus and Espacenet by using as query: biocatalysis, chiral and pharmaceutical or drug ‐ last update May 20, 2022)

About 57% of active pharmaceutical ingredients (APIs) are chiral molecules, and accordingly, most of the chiral drugs are marketed in homochiral form.^1^, ^2^ In the past, chiral drugs were usually developed as racemic mixtures since separation into the individual enantiomers or the synthesis of homochiral compounds was difficult to achieve. The introduction of strict rules on the use of racemates set by drug regulatory agencies has been an engine for the development of technologies able to produce single enantiomers on large scale.^3^ Accordingly, for the past 30 years, both academia and pharmaceutical companies have been working to develop efficient procedures for obtaining homochiral compounds in good yields and with excellent enantiomeric excess.^4^ Among the approaches available so far, in the present review, we focus on biocatalysis, that is, the use of isolated enzymes from biological sources or whole cells for performing organic reactions.^2^, ^5^, ^6^ The idea that the use of enzymes as catalysts is just an academic speculation belongs to the past, and biocatalysis is now recognized as a highly valuable tool for cost‐effective and sustainable pharmaceutical manufacturing.^7^, ^8^ Several advantages are associated with biocatalysis,^5^ such as the use of biodegradable enzymatic catalysts, the limited number of synthetic steps, the mild reaction conditions, and the high enantio‐, chemo‐ and regioselectivities.^6^, ^9^, ^10^, ^11^ Nevertheless, its potential is not yet fully exploited. Enzyme‐catalyzed reactions possess many appealing characteristics for the synthesis of chiral intermediates, because reactions can be carried out at room temperature and atmospheric pressure, thus limiting, or avoiding, isomerization, racemization, epimerization, and rearrangement.^12^, ^13^ For all these reasons, the development of biocatalytic processes to obtain chiral building blocks is of particular interest in the pharmaceutical field.^14^ Over the years, the interest in biocatalysis to obtain pharmaceutical intermediates has dramatically increased, as evidenced by the number of publications and patents. As highlighted in Figure 1, from the beginning of the 2000s, applications of biocatalysis in the pharmaceutical field exploded with growing interest up to the present days.

The use of biocatalysis in the pharmaceutical industry for the production of intermediates, enantiomerically pure building blocks, and APIs is expanding. With the expansion of pharmaceutical industries, the demand for biopharmaceuticals is also increasing, and this acts as a driver for the biocatalysis and biocatalysts market.^15^ This trend is confirmed by the increasing number of publications and patents owned by pharmaceutical companies for biocatalytic approaches. A few examples of patents include (i) a carboxylesterase engineered enzyme disclosed by GlaxoSmithKline for esters and amines amidations to produce a key intermediate in the synthesis of indazole drug candidates; (ii) the use of a D‐threonine aldolase for the kilogram‐scale preparation of β‐hydroxy‐α‐amino acid for a drug candidate reported by Bristol‐Myers Squibb; (iii) the use of a deoxyribose‐5‐phosphate aldolase as part of a multienzyme process for the preparation of the human immunodeficiency virus (HIV) investigational drug Islatravir published by Merck and Codexis (vide infra)^16^; and (iv) the stereoselective hydrolysis of a racemic mixture of N‐protected cis‐4‐amino‐cyclopent‐2‐en‐1‐ol (cis‐8) carried out using lipase B from *Candida antarctica* patented by Beijing University of Chemical Technology.^17^

Although the high potential of biocatalysis for obtaining enantiopure products is now well recognized, this technique is still not widely exploited in industry, mainly because enzymes are conceived as biological agents rather than chemical reagents. The aim of this review is to highlight the potential of biocatalytic approaches for synthesizing chiral pharmaceutical intermediates. After facing the main challenges related to the use of enzymes (i.e., immobilized enzymes, including nanobiocatalysts, whole‐cell catalysts) we will focus on the potential application of biocatalysis in pharmaceutical industry with an emphasis on green aspects and the chiral synthesis of drugs and/or their intermediates. We will underline how biocatalytic transformations may revolutionize the way for enantiopure APIs production.

## 2 TYPES OF BIOCATALYSTS

### 2.1 Immobilized biocatalysts

Since the early 2000s, the scientific community has developed diverse enzyme collections and has also enabled techniques to increase enzyme process performance, thus leading biocatalysis to be a technology widely applied.^18^ Within this context, enzyme immobilization played a crucial role, as discussed here below.

Enzyme immobilization is a powerful technique for enhancing enzyme catalytic properties, that is, increasing their activity, stereoselectivity or stability, and operational performances, such as recovery and reusability of costly enzymes, in many applications a key aspect for economic viability.^19^ Immobilization strategy allowed development of biocatalytic processes feasible, making innovations in chiral pharma.^20^ Several procedures have been reported, comprising integrating biocatalysts into a variety of industrially interesting processes ranging from the manufacture of small, chiral building blocks to the synthesis of more complex pharmaceutical intermediates. Accordingly, the application of biocatalysis in the pharmaceutical industry is expanding, and there is a need for easy‐to‐use biocatalysts to meet the demands of industrial processes.^21^ The immobilization of enzymes allows the development of biocatalysts with improved catalytic characteristics in a wide range of operating conditions.^22^ More in detail, immobilization consists of fixing enzymes to or within solid supports, creating a heterogeneous immobilized enzyme system. In such a way, their natural mode in living cells, where most of them are attached to cellular cytoskeleton, membrane, and organelle structures, is mimicked. Solid supports generally stabilize the three‐dimensional structure of the enzymes and, as a consequence, maintain their catalytic activities. Enzyme immobilization was initially designed to allow an easy enzyme recovery from aqueous media.^23^, ^24^, ^25^ Afterwards, it has been evidenced that immobilization facilitates downstream processing and continuous operation and allows to face many other enzyme limitations, like enzyme activity, stability, or purity, and tunes enzyme selectivity or specificity.^26^, ^27^, ^28^ Accordingly, immobilization objectives are nowadays far away from the initial mere enzyme recovery, and a proper immobilization method that optimizes the different enzyme features may become a critical step in the design of an industrial biocatalyst.^29^, ^30^, ^31^

**FIGURE 2:**
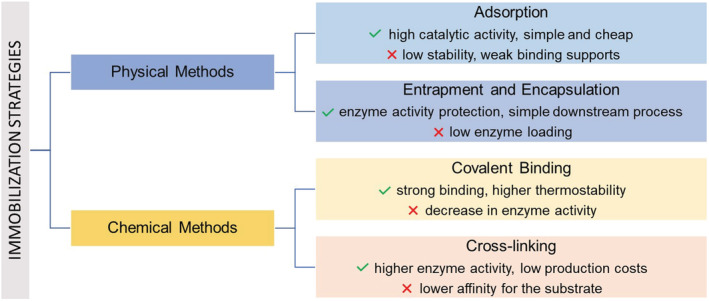
Enzyme immobilization methods, pros, and cons

Over the years, numerous enzyme immobilization methods have been set up, depending on the nature of both enzymes, and supports and can be broadly grouped into physical or chemical immobilization methods (Figure 2).^32^, ^33^

*Physical immobilization* of enzymes can be achieved with hydrophobic and Van der Waals ionic or affinity interactions. This strategy aims to maintain the structural integrity of the enzyme, and thus maximizing catalytic activity.

Generally, the physical immobilization technique includes the entrapment and encapsulation methods. The entrapment methods consist of inclusion of enzymes in a polymeric network, typically organic or inorganic matrices, such as polyacrylamide and silica sol–gel, or a membrane device such as a hollow fiber or a microcapsule. These matrices are usually utilized for the entrapment of whole cells and soluble enzymes.^34^ The enzyme encapsulation foresees that the enzyme unit is separated from the reaction environment by an organic or an inorganic polymer.^34^ Several examples reported the use of phospholipid vesicles (liposomes) or synthetic polymers (polymersomes), or recently the use of nanoparticles.^35^ Compartmentalizing enzymes, these systems mimic a similar biological environment increasing their catalytic efficiency and stability.

*Chemical immobilization* is based on the enzyme attachment to solid matrices or supported by covalent interactions.^36^ Various types of material (organic, inorganic, hybrid, or composite) can be used as supports for enzyme immobilization, and each material may influence the properties of the biocatalytic system.^37^ Enzymes chemical immobilization can also be allowed in the absence of a support by the formation of enzyme aggregates via cross‐linking of enzyme molecules.^38^ In this latter case, enzyme units are covalently connected to each other by cross‐linkers.^39^ The great advantages of this approach include high enzyme activity and stability and low production costs owing to the exclusion of an additional (expensive) carrier.^38^ Recent examples of cross‐linking methods have been investigated with the aim to develop and improve efficiency of enzymatic parameters such as pH, temperature, kinetic properties, operational stability, and recyclability. For a more in‐depth and detailed definition, see references.^32^, ^33^, ^34^, ^35^, ^38^ Noteworthy examples of enzyme immobilization and their applications to the production of chiral API are reported in Table 1.

**TABLE 1:** Examples of industrial applications using immobilized enzymes for the production of different APIs

Immobilized enzyme	Type of immobilization	Homochiral API
Galactose oxidase (evolved) from *Fusarium graminearum*	Affinity on Nuvia IMAC resin charged with nickel	Islatravir^16^
Lipase B from *Candida antarctica*	Adsorption on octadecyl polymethacrylate resin	Odanacatib^40^
Lipase B from *Candida antarctica*	Adsorption on methacrylate/divinylbenzene copolymer	Sofosbuvir^31^
Penicillin G amidase	Covalent on epoxy or amino methacrylate polymer	Amoxicillin/Ampicillin^41^
Transaminase	Adsorption	Sitagliptin^42^

### 2.2 Nanobiocatalysts

An improvement of the enzyme immobilization strategy foresees immobilization on solid nano supports: this technique is known as nanobiocatalysis.^43^ Nanobiocatalysts (NBCs) are biomaterials originated by the consolidated union of advanced biotechnology and nanotechnology. In the last decade, there was an increasing interest in the use of nanobiocatalytic tools because of their stability to improve enzyme stability, function, and efficiency.^43^ A wide range of biomaterials may be used for the development of innovative NBCs with various applications in biological, medical, and pharmaceutical fields.^14^, ^44^

Nanosupports are functionalized nanocarriers developed by surface modification of several nanomaterials (nanotubes, nanocages, nanocomposites, etc.) with a variety of other materials such as noble metals, metal oxides, and metal–organic frameworks. To produce NBCs with potential activity and stability, the immobilization of enzymes is achieved by several attachment methods that generate interactions with an inert support material, so that enzymes maintain full or most of their biocatalytic performance.^14^, ^38^

Physical and chemical immobilization techniques can be used to produce NBCs with potential activity and stability.^14^ However, physical adsorption is commonly preferred when working with NBCs with abundant charged surface chemical groups and lead to the formation of either hydrophobic or hydrophilic interactions, resulting in stabilization of the NBCs.^43^ NBCs can be applied in various nanobiocatalytic systems, such as batch, multi‐enzymatic or reactor‐based as well.^14^

**FIGURE 3:**
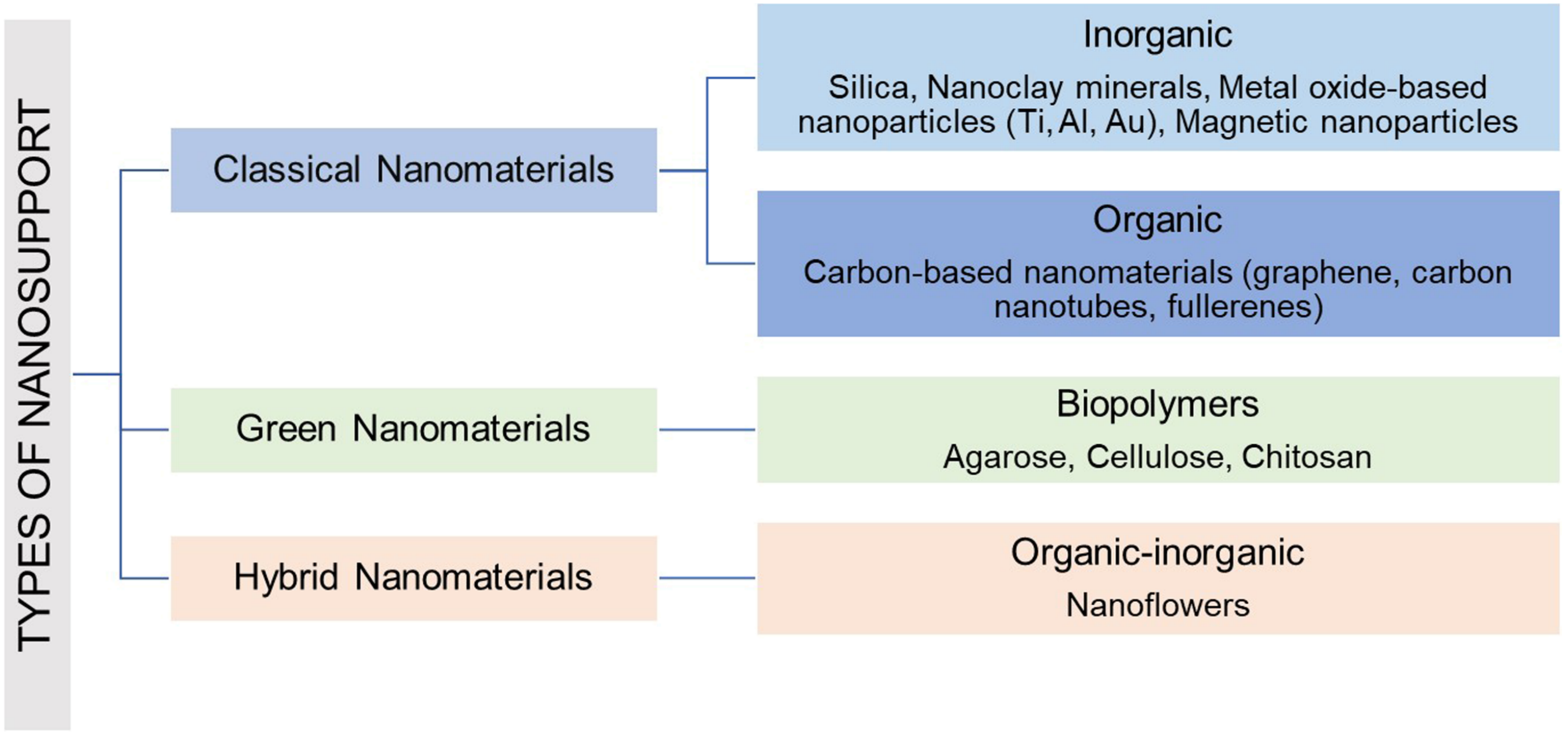
Nanomaterials for biocatalytic processes

Nanomaterials developed can be classified as shown in Figure 3.^44^

*Classical nanomaterials* can be further divided into inorganic and organic according to the type of support (metal based vs carbon based). Inorganic nanosupports are low‐cost materials with high availability in nature, excellent mechanical and thermal stability, and microbial resistance.^45^ Organic supports, such as fullerenes and carbon nanotubes, can be functionalized and further hybridized with other nanomaterials in order to develop advanced nanocomposites with better solvent solubility or biocompatibility.^14^

*Green nanomaterials* represent a new trend in the development of innovative NBCs. The exploitation of these biomaterials substantially facilitates the immobilization of enzymes compared to conventional methods. Particularly, biopolymers (such as cellulose and chitosan) are an attractive alternative to the conventional materials and are able to improve work performance, such as the enzyme catalytic activity.^46^ Furthermore, *organic–inorganic hybrid nanoflowers* (NFs), a class of nanomaterials with flower‐like structure, represent an additional attractive candidate for the applications in biocatalysis. In several studies, NFs have exhibited greater catalytic activities compared to those of soluble enzymes.^47^, ^48^ Their application to the synthesis of chiral intermediates, drug candidates, and APIs is expanding.

**FIGURE 4:**
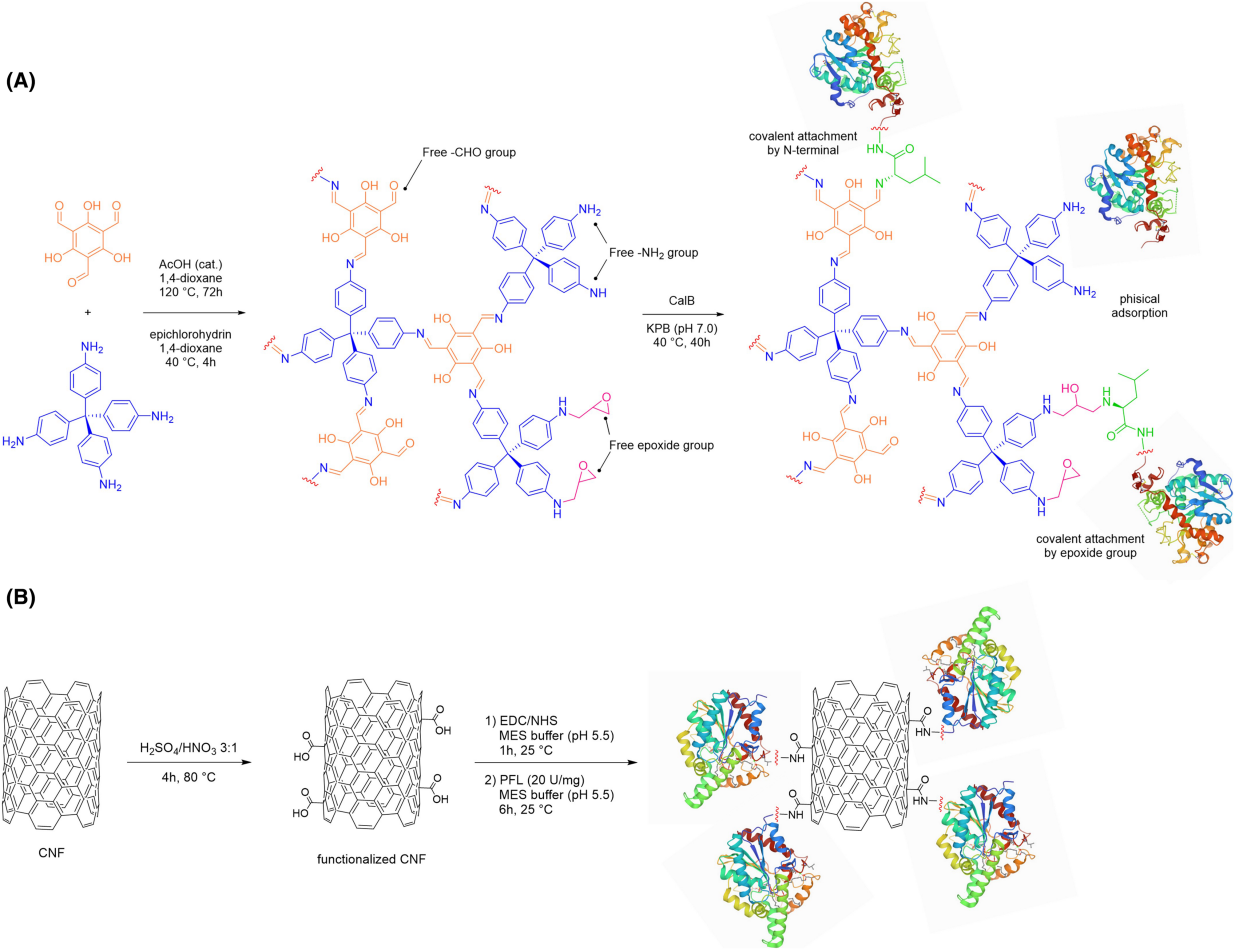
(A) Schematic representation of COF synthesis, functionalization, and enzyme immobilization; (B) Example of a strategy for enzyme immobilization on CNF. COF, covalent organic framework; CNF, carbon nanofiber

Recently, Oliveira et al. reported on the immobilization of *Candida antarctica* lipase B (CaL‐B) on a covalent organic framework (COF/PPF‐2) to achieve a highly valuable NBC. In fact, lipases are useful tools to produce homochiral compounds, albeit soluble biocatalysts could show some issues.^49^ Accordingly, the authors exploited different methodologies of immobilization of the CaL‐B on the organic framework. Through the use of this platform, they demonstrated the increase of the enzymatic activity, an improved thermal stability and reusability of the biocatalyst through several cycles of reactions (Figure 4A).^49^ Similarly, Dwivedee and colleagues immobilized *Pseudomonas fluorescens* lipase on carbon nanofiber to obtain a highly performing biocatalyst for the synthesis of enantiopure (*R*)‐carboetomidate, an etomidate analog used as anesthetic. This approach improved fourfold the efficiency of the NBC compared to the soluble enzyme. Moreover, the authors reported that the NBC showed an increase in thermal stability (up to 85 °C) along with a high reusability (Figure 4B).^50^

### 2.3 Whole‐cell biocatalysts

The use of purified (recombinantly expressed) enzymes both in soluble or immobilized form can be an expensive technology. Purified enzymes are roughly 10‐fold more expensive than whole cells.^48^ Moreover, subsequent catalyst immobilization can add even more costs to the process because of the extra costs of the carrier material (that can be particularly impactful) as well as to the additional operational procedures required. Thus, the use of whole cells as biocatalysts can represent a valuable alternative to the use of crude, purified, or immobilized enzyme preparations. In addition to being the cheapest form of catalyst formulation, whole cells are usually employed to mediate cofactor‐dependent reactions, as cells can easily supply and regenerate the cofactors needed for biotransformations, making an external and expensive cofactor addition obsolete.^51^ Furthermore, residual cell wall compounds act in a protective manner, thus enabling enzyme applications under harsh reaction conditions or in un‐conventional (non‐aqueous) reaction media.^51^ Despite their intrinsic stability, whole cells can also be immobilized in order to facilitate their recovery and recyclability, thus lowering the operational costs of the process.^52^ Whole cells can allow the combination of different enzymes into multi‐step cascade reactions to access complex and valuable products from inexpensive starting substrates. Co‐expressing multiple enzymes in one cell enhances reaction efficiency because of the close proximity of biocatalysts in the cellular environment that can act in consecutive steps within a confined space. However, co‐expression of multiple proteins may lead to a high metabolic burden during cell growth, which can result in poor overexpression and thus impaired catalytic activity.^53^ Despite the advantages outlined so far, some drawbacks remain for whole‐cell catalysts. Often whole‐cell biocatalysts can show lower selectivity compared to soluble purified enzymes because of unwanted side reactions that can be mediated by other competitive enzymes present in the cells. Another drawback when using whole‐cell biocatalysts is substrates/products mass transfer limitation because of the presence of cell membrane that can prevent substrate and product diffusion in and out from the cell. Also, because of permeability barriers imposed by cell envelopes, whole‐cell catalyzed reactions are reportedly 10‐ to100‐fold slower than reactions catalyzed by soluble enzymes. However, in some studies, the reaction catalyzed by whole cells was accelerated by reducing the membrane permeability barrier using molecular engineering approach.^54^ Lastly, some regulatory concerns might be raised when whole‐cell catalysts are used in the production of APIs, because of possible contamination of the final product.

Nevertheless, several strategies have been developed and are currently under investigation to overcome the limitations related to whole‐cell biocatalysis, including segmented flow tools, synthetic biology, and metabolic engineering toolboxes. Hence, numerous applications of whole‐cell reactions can be found in established pharmaceutical processes.^55^ Most popular applications of whole‐cell biocatalysts include oxidoreductases and aminotransferases for the synthesis of chiral alcohols, amino alcohols, amino acids, and amines. Monooxygenases in the whole cells have been used in enantioselective and regioselective hydroxylation, epoxidation, and Baeyer–Villiger reactions. Dioxygenases have been used in the synthesis of chiral diols. In addition, hydrolytic enzymes have been applied as whole‐cell biocatalysts for the resolution of a variety of racemic compounds and in the asymmetric synthesis of enantiomerically enriched chiral compounds.^56^ Some examples of whole‐cell biocatalyzed processes used by the pharmaceutical industry to prepare chiral intermediates for the production of final drug substances are given in Table 2 and discussed in the next paragraph.

**TABLE 2:** Examples of industrial application using wholecells as biocatalyst

Whole‐cell biocatalyst	Type of biocatalyst	Homochiral API
*Lactobacillus kefri P2*	Not immobilized cells	Bufuralol^57^
Recombinant *Escherichia coli*	Immobilized cells	Telaprevir^58^
Recombinant *Escherichia coli*	Not immobilized cells	AZD1480^59^
*Erwinia herbicola*	Not immobilized cells	L‐DOPA^60^
*Streptomyces carbophilus*	Not immobilized cells	Pravastatin^61^

## 3 BIOCATALYTIC APPROACHES FOR CHIRAL DRUGS PREPARATION IN PHARMACEUTICAL INDUSTRY

As discussed above, there is a growing interest toward the use of biocatalysis to produce homochiral drugs, and its use, when possible, is now considered a winning strategy not only from an economic standpoint but also in terms of sustainability and environmental impact. Hence, biocatalysis is now added among other well‐established tools in green chemistry, such as organocatalysis, multicomponent reactions, and solvent‐free synthesis, just to cite a few examples. A broader view on the use of such approaches in the synthesis of pharmaceuticals is beyond the scope of this work and can be found in other excellent reviews on the topic.^62^, ^63^, ^64^ Herein, we focus on examples, selected from recent literature, that show the successful use of biocatalysis in the synthesis of chiral APIs.

**FIGURE 5:**
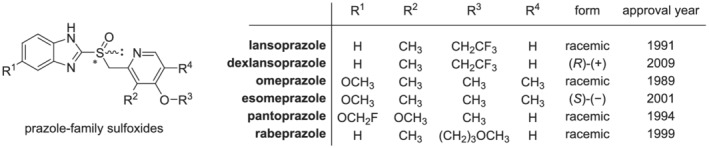
Chemical structures of commercially available prazole‐family PPIs, chiral form (racemic/enantiopure), and approval year thereof by European or USA regulatory agencies. PPIs, proton pump inhibitors

Proton pump inhibitors (PPIs), largely used for clinical treatment of gastrointestinal diseases, are an important class of drugs, included by the World Health Organization in the list of essential medicines.^65^ Most of these drugs have been originally approved and marketed between the 1980s and 1990s as racemates, but later the “old” racemates have been re‐evaluated, according to the “chiral switch” process, that is, the replacement of a chiral drug used in the form of a racemate with its eutomer, characterized by better pharmacodynamic, pharmacokinetic, and pharmacological profiles (Figure 5).

**SCHEME 1:**
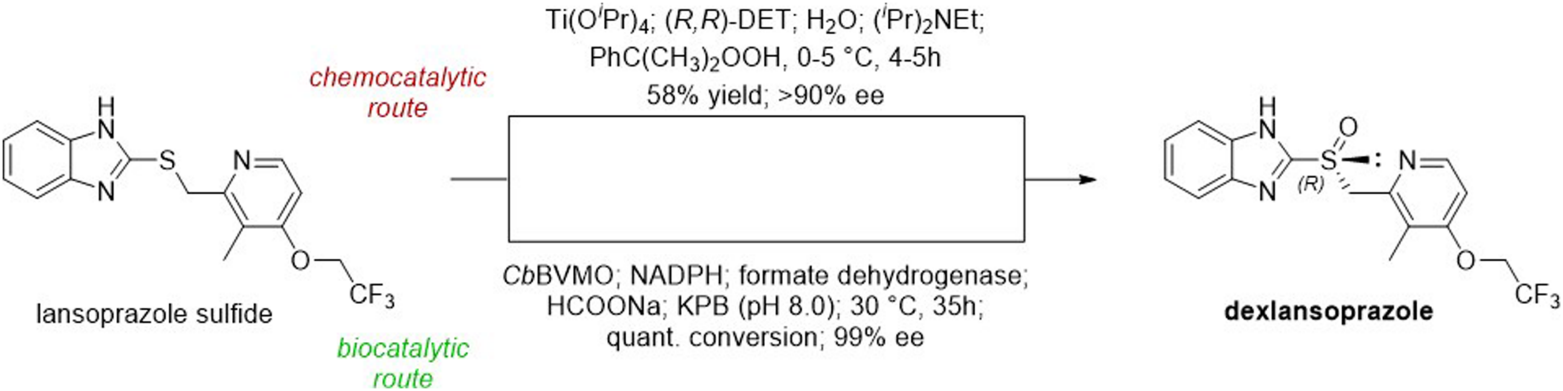
Comparison between chemo‐ and biocatalytic approaches for the synthesis of (*R*)‐lansoprazole (dexlansoprazole) from the sulfide precursor

PPIs belonging to the prazole family are characterized by the presence of a chiral sulfur atom (as shown in Figure 5). The most straightforward and convenient approach to produce homochiral sulfoxides is asymmetric sulfoxidation of prochiral sulfides. A biocatalytic approach for the synthesis of dexlansoprazole, which was recently reported in literature, is discussed herein as an example to highlight the advantages over the previously developed chemo‐catalytic route for the synthesis of this class of chiral sulfoxides (Scheme 1).^66^ Dexlansoprazole is a PPI specifically developed for anti‐ulcer activity by TAP pharmaceuticals Ltd. It is the (*R*)‐enantiomer of lansoprazole, with a higher therapeutic efficacy with respect to the (*S*)‐enantiomer.

Chemocatalysis has been successfully used by Raju and collaborators for the preparation of dexlansoprazole from lansoprazole sulfide, exploiting a chiral titanium complex under modified Kagan's oxidation conditions.^67^ Albeit good results were obtained, in terms of yield and enantiomeric excess, the protocol suffers significant drawbacks such as the cost of the reactants, the tight control of the reaction conditions required, laborious downstream operations, and the considerable amount of waste generated. In particular, the removal of the unwanted (*S*)‐enantiomer during the fabrication process is quite complicated and requires a large amount of solvents, leading to a low yield of the final product. Accordingly, Liu and co‐workers explored the viability of biocatalysis for a more efficient and environmentally friendly production of dexlansoprazole, with the use of molecular oxygen as oxidant.^66^ Of note, only a few native biocatalysts can effectively be used for the enantioselective oxidation of prazole‐family sulfides, mainly because of their low activity. However, starting from previous experimentations in this field, and exploiting the sequence of *Bo*BVMO (Baeyer‐Villiger monooxygenase from *Bradyrhizobium oligotrophicum*) as a probe for genome mining, the authors identified a new BVMO from *Cupriavidus basilensis* (*Cb*BVMO) with optimal activity toward the precursor lansoprazole sulfide (99% ee and 100% conversion over 35 h). Moreover, the authors verified the efficacy of this new biocatalyst on other three prazole sulfides (i.e., omeprazole, pantoprazole, and rabeprazole) and found it to give excellent results, highlighting the great potential of BVMOs in pharmaceutical industry.^66^

**SCHEME 2:**
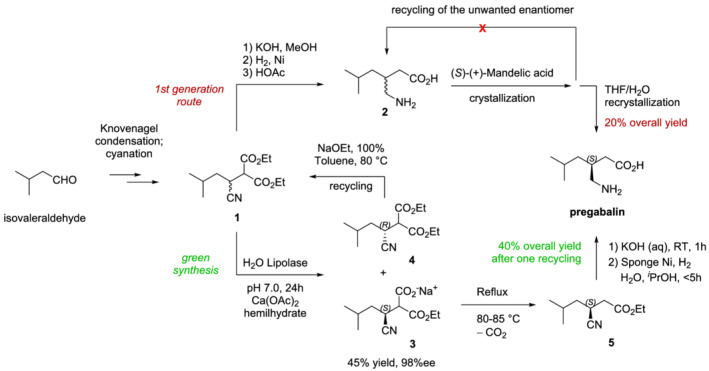
Comparison between first generation and green syntheses of pregabalin

The green synthesis of pregabalin represents another example of success in the use of biocatalysis to reduce the environmental impact of the production of chiral pharmaceutical intermediates.^68^ Pregabalin is the active ingredient of Lyrica, used for the treatment of various disorders of the central nervous system, including neuropathic pain, epilepsy, and anxiety. The first‐generation synthesis and the new green synthesis of pregabalin have a common cyano‐diester intermediate (compound **1** in Scheme 2), which can be easily obtained starting from isovaleraldehyde, after Knoevenagel condensation and cyanation. Then, the original route involves hydrolysis, Ni‐catalyzed hydrogenation, and decarboxylation to give the racemic γ‐amino acid. This can be resolved with a stoichiometric amount of (*S*)‐mandelic acid, followed by re‐crystallization in THF/H_2_O in order to obtain pregabalin, with an overall process yield of about 20%. This process suffered from low atom economy, high organic waste generation, and the impossibility to recycle the unwanted enantiomer.^69^ Instead, using Lipolase‐catalyzed resolution of the cyano‐diester **1**, the desired (*S*)‐mono acid enantiomer (compound **3**) can be obtained with high‐resolution yields (45%) and high enantioselectivity (98% ee). Afterwards, heat‐promoted decarboxylation, hydrolysis, and hydrogenation yield enantiopure pregabalin. Moreover, the undesired (*R*)‐enantiomer corresponding to compound **4** can be readily racemized and recycled, increasing the process yield up to 40% after just one recycling step. Additionally, it must be noted that, beside the excellent performance in terms of yield and enantioselectivity, the solvent required to conduct all the three steps from **1** is water. This contributes to making the biocatalytic process greener than the first‐generation synthesis, with a drastic decrease of the *E‐*factor from 86 to 17.^68^

**SCHEME 3:**
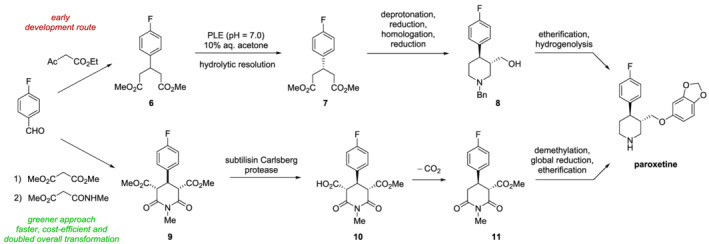
Synthesis of paroxetine. Comparison between the early development route and the more recent green synthesis

Likewise, the synthesis of paroxetine, a drug widely used for the treatment of depression, can be highly improved by biocatalysis, benefitting from similar advantages to pregabalin. Different attempts were made to improve the original synthesis, which was particularly tortuous and inefficient.^70^ A first attempt to ameliorate it was experimented by GlaxoSmithKline as schematized in Scheme 3 (development route): starting from 4‐fluoro benzaldehyde, the synthesis exploits the biocatalytic desymmetrization of compound **6**, mediated by pig liver esterase (PLE), as an efficient strategy to introduce the first chiral center. However, the overall route involved several synthetic steps and did generate significant amounts of waste. Accordingly, a greener approach was later developed, which exploits the same starting material. As depicted in Scheme 3, after Knoevenagel condensation, Michael addition, and intramolecular cyclization the meso‐diester **9** is obtained. This key intermediate is subjected to a desymmetrization catalyzed by subtilisin Carlsberg, which belongs to the family of serine endopeptidases isolated from *Bacillus subtilis*. Then, decarboxylation of **10** leads to the single enantiomer with a good yield (84%) and high ee value (94%). Finally, a reduction step is necessary to obtain (−) Paroxol, the intermediate preceding paroxetine. Thus, redesigning the synthesis with an enzymatic desymmetrization, the yield of the new process was almost doubled with respect to the previous resulting in a greener, shorter, and cost‐efficient approach.^71^

**SCHEME 4:**
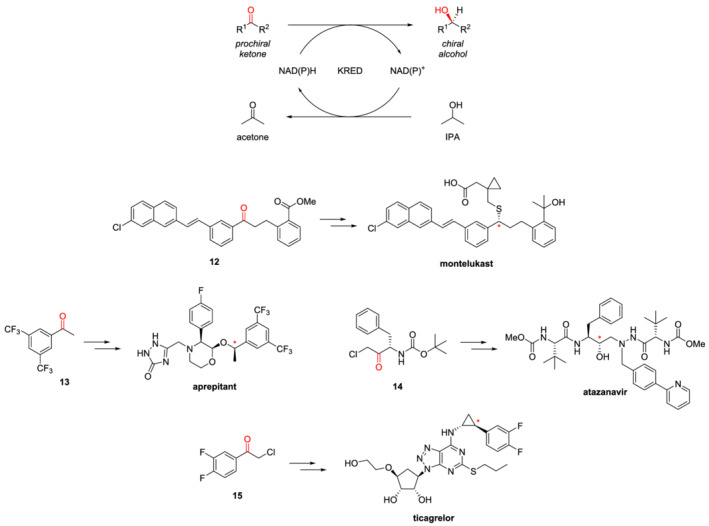
Enantioselective reduction of ketones catalyzed by KRED, with NAD(P)H recycling system (top); examples of the use of KRED in the synthesis of APIs (bottom). The carbonyl that is selectively reduced is colored in red in the precursors, whereas the stereocenter that is thus introduced in the final product is marked with a red asterisk.

A broadly useful application of biocatalysis in the pharmaceutical industry is the use of ketone reductases (KREDs) to transform prochiral ketones into chiral alcohols. Given the importance of this functionality – both as pharmacophoric group to engage specific drug‐target interactions and as a versatile functionality for further modifications – KREDs can be exploited in the preparation of simple building blocks and key intermediates, as well as final products and for late‐stage modifications. The synthesis of montelukast is currently one the most notable and well‐established example of the use of a KRED in the large‐scale preparation of an API.^72^ Montelukast is a leukotriene receptor antagonist used to control the symptoms of asthma and seasonal allergies. The original route developed by Merck involves the enantioselective reduction of ketone **12** to the corresponding (*S*)‐alcohol, which then undergoes a S_N_2 reaction with a thiol to give the final product with the desired (*R*) configuration. This process used an expensive chemical reducing agent, that is, (−)‐DIP‐Cl, and generated large amounts of waste. In the search for a more efficient and sustainable protocol, Codexis developed a biocatalytic reduction directed by the engineered KRED CDX‐026, obtaining the product in >95% yield and >99.9% ee (Scheme 4).^72^ More recently, Raynbird and colleagues explored the possibility to expand the use of KREDs to the preparation of other API, for which biocatalysis had not been taken into consideration yet.^73^ The precursor ketones of eight different drugs were acquired and screened against more than 400 commercially available KREDs from different suppliers. The authors identified a single KRED with high activity and selectivity for ketones **13**–**15**, key intermediates in the synthesis of aprepitant, which is a neurokinin‐1 (NK‐1) receptor antagonist used as antiemetic adjunct in chemotherapy, atazanavir (an antiretroviral agent used to treat HIV/acquired immunodeficiency syndrome) and ticagrelor, a platelet aggregation inhibitor for the prevention of thrombosis in patients with the acute coronary syndrome (Scheme 4). These were then subjected to reaction optimization, which easily allowed the scale‐up to 1 L scale in high conversion and isolated yield while retaining selectivity of >99.5% ee.^73^ One key aspect to be considered when developing a KRED‐catalyzed process is the need for the expensive co‐factor NAD(P)H. This can be recycled by exploiting the reverse reaction (i.e., oxidation of a cheap alcohol to the corresponding ketone), in order to make the biocatalytic process competitive and practical. Two main options are available as co‐factor recycling systems: isopropanol (IPA)/KRED or glucose/glucose dehydrogenase. The first has the great advantage that does not require any additional enzyme, since the KRED can catalyze the reverse reaction, oxidizing IPA to acetone. This can be easily removed from the reaction medium by distillation, thus driving the coupled reaction forward to high conversion rates. All the KRED‐catalyzed reactions described above exploit this recycling system, with the additional benefit of IPA serving also as co‐solvent, thus providing highly efficient and environmentally friendly processes.^72^, ^73^

**SCHEME 5:**
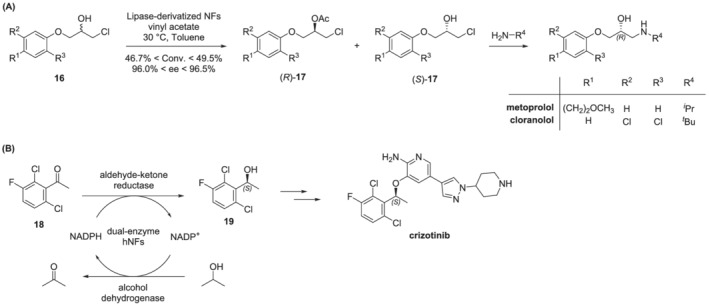
(A) Kinetic resolution of racemic intermediates of two β‐blocker drugs; (B) enantioselective reduction of a prochiral ketone for the synthesis of crizotinib.

As previously stated, among most recent advances in the biocatalysis field, NBCs deserve a particular mention. Because of their stability, bioprocessing efficiency, and downstream recovery, NBCs represent a class of promising nanomaterials that embrace enormous applications in the pharmaceutical industry. Recently, the *Burkholderia cepacia* lipase was efficiently immobilized in a new hybrid NFs that displayed a marked improvement in the kinetic resolution of racemic intermediates of two β‐blocker drugs (i.e., metoprolol and cloranolol), as outlined in Scheme 5a.^74^ Finally, an innovative approach that use dual‐enzyme@CaHPO_4_ hybrid nanoflowers (hNFs) was recently utilized to efficiently catalyze the synthesis of the chiral compound (*S*)‐1‐(2,6‐dichloro‐3‐fluorophenyl) ethyl alcohol **19**, an important intermediate of the anti‐cancer crizotinib (Scheme 5b).^75^ Furthermore, the use of this innovative nanosupport improves enzymatic activity, thermal stability, and recycling cycles of synthetic reaction (up to 16) and may be applicable also in multi‐enzyme systems.

Thus, these nanomaterials can be versatile host platforms for the immobilization of a plethora of enzymes that could efficiently improve their catalytic performance and potentially be used as efficient tools for heterogeneous biocatalytic transformations in large‐scale applications.

**SCHEME 6:**
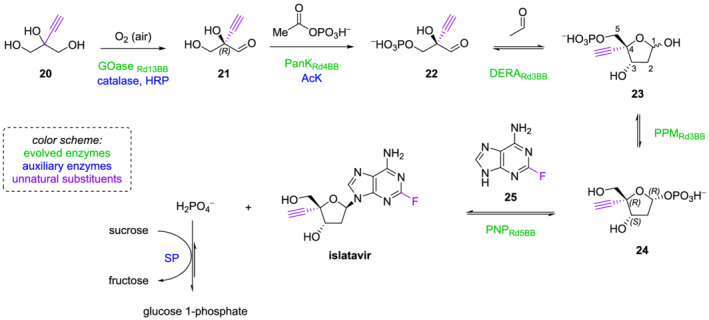
In vitro biocatalytic cascade synthesis of islatravir

Another breakthrough in the field of biocatalysis is represented by the in vitro biocatalytic cascade synthesis of islatravir: a nucleoside reverse transcriptase translocation inhibitor under clinical investigation for the treatment of HIV. This molecule contains three stereogenic centers with defined absolute configuration. Different syntheses have been developed to access islatravir, but many of them had important flaws, such as the need for several steps (including protections and deprotections) and the challenges in the control of anomeric stereochemistry.^76^, ^77^, ^78^ In 2019, Huffman and collaborators at Merck developed an elegant biocatalytic cascade approach to the synthesis of islatravir.^16^ This takes advantage of the bacterial nucleoside salvage pathway, which degrades purine 2′‐deoxyribonucleosides into glyceraldehyde 3‐phosphate and acetaldehyde using three enzymes: purine nucleoside phosphorylase (PNP), phosphopentomutase (PPM), and deoxyribose 5‐phosphate aldolase (DERA). Because the reactions in the salvage pathway are reversible, the authors envisaged the engineering of these enzymes to make them work backwards on unnatural substrates, as a promising strategy to efficiently obtain the target molecule from simple starting materials. As reported in Scheme 6, an evolved galactose oxidase (GOase) from *Fusarium graminearum* is used to perform a desymmetrizing oxidation of 2‐ethynylglycerol, yielding the desired (*R*)‐aldehyde **21** with high selectivity. For preparative applications, this transformation requires two additional redox enzymes: a catalase to disproportionate the H_2_O_2_ formed as byproduct, and a peroxidase to maintain the correct Cu oxidation state. A commercially available catalase from *Bos taurus* and a horseradish peroxidase from *Amoracia rusticana* served these purposes. Then, an engineered pantothenate kinase (PanK) from *Escherichia coli* was used for the selective phosphorylation of the primary alcohol of **21**, employing acetyl phosphate as a cheap phosphate source. The PanK was co‐immobilized with an acetate kinase (AcK) from *Thermotoga maritima*, necessary for the regeneration of the cofactor adenosine triphosphate. The obtained product **22** is then subjected to aldol reaction with acetaldehyde, which triggers the subsequent cyclization to yield the unnatural sugar **23**. This step was catalyzed by a DERA originally isolated from *Shewanella halifaxensis* and evolved to increase its stability under high acetaldehyde concentrations. The new C–C bond was formed with excellent stereoselectivity (> 99% de), and the kinetic selectivity of the biocatalyst toward the (*R*)‐enantiomer of aldehyde **21** further increased the enantiomeric purity of the product. The last two synthetic steps involve the conversion of the 5‐phosphate sugar **23** to its 1‐phosphate isomer **24** and the glycosylation of the adenine analog **25**. These were respectively catalyzed by a PPM and a PNP, both evolved from *E. coli*. Because the PPM‐catalyzed reaction is an equilibrium favoring the thermodynamically more stable 5‐phosphate **23**, the authors aimed at performing the two transformations simultaneously. However, the hydrogen phosphate byproduct that originates in the last step is a known inhibitor of PPM. To overcome these potential limitations, sucrose phosphorylase (SP) and sucrose were added to the reaction mixture to convert the free phosphate to glucose 1‐phosphate and pull forward the entire equilibrium. Hence, by fine tuning the reaction conditions of each step and exploiting properly engineered enzymes, the authors were able to develop a fully biocatalyzed, in vitro cascade synthesis of islatravir. The process involves five evolved enzymes to perform, in reverse and on non‐natural substrates, the key transformations of the nucleoside salvage pathway, and four auxiliary enzymes. By this approach, enantiopure islatravir can be obtained starting from simple achiral building blocks in 51% overall yield. In addition, the use of mild conditions and a single aqueous solution without isolation of intermediates is a great advantage in terms of efficiency and sustainability.^16^

4

As discussed above, using whole‐cell systems as biocatalysts can, in some circumstances, bypass the necessity for cell lysis and enzyme purification, cutting the cost of synthetic processes.^79^, ^80^

**SCHEME 7:**
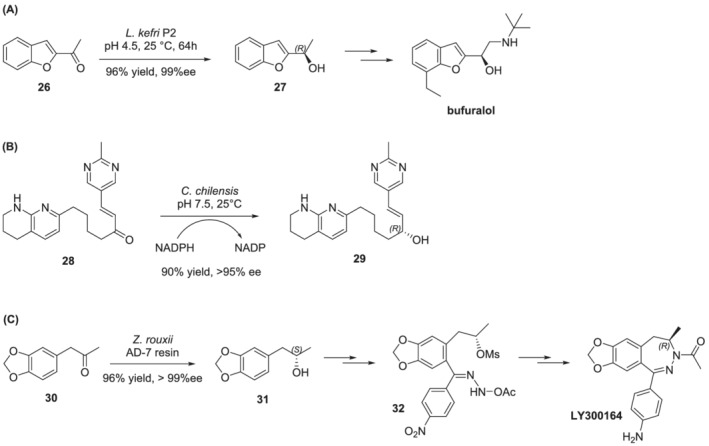
Examples of the application of whole‐cell biocatalysts in the synthesis of pharmaceuticals

One example of a whole‐cell system, *Lactobacillus kefri P2*, catalyzes the asymmetric reduction of prochiral ketones to chiral secondary alcohols that can be used in the synthesis of pharmaceuticals such as bufuralol (a potent and non‐selective β‐blocker), the antiarrhythmic amiodarone, and the coronary vasodilator benziodarone (Scheme 7).^57^ Similarly, the whole‐cell approach proved to be successful in the synthesis of an integrin receptor antagonist for the inhibition of bone resorption and the treatment of osteoporosis. A pilot scale whole‐cell process was developed by Merck for the enantioselective reduction of prochiral α,β‐unsaturated ketone **28** to (*R*) allylic alcohol **29** using *Candida chilensis*.^81^ This resulted in a valuable and greener alternative to chemical reduction routes.

Finally, compound LY300164, a 2,3‐benzodiazepine which has been under clinical evaluation for treatment of epilepsy and Parkinson's disease, also underwent a synthetic optimization process that exploits whole‐cells biocatalysis. The target molecule was prepared from (*S*)‐4‐(3,4‐methylene‐dioxyphenyl)‐2‐propanol **31**, which in turn was derived from 3,4‐methylenedioxyphenylacetone **30** by a dehydrogenase from the yeast *Zygosaccharomyces rouxii* via the whole‐cell biotransformation process (Scheme 7).^56^

## 5 CONCLUSIONS AND FUTURE PERSPECTIVES

As discussed above, biocatalysts show several advantages in the preparation of chiral intermediates for the synthesis of homochiral drugs, because of their high selectivity, high activity under mild reaction conditions, and their biodegradable and non‐toxic nature. Over the past few decades, many of the enzyme‐catalyzed processes have been successfully established and scaled up and accordingly biocatalysis, with both immobilized‐enzyme and whole‐cell systems, is becoming a universal methodology for the biosynthesis of enantiomerically pure compounds in laboratory scale as well as in industrial scale.^2^ Following the rapid growth of nanobiotechnology and nanotechnology, the synergistic interactions between these two technologies have resulted in the cutting edge of biocatalysis: nanobiocatalysis. Defined as a new frontier of emerging nanosized material support in enzyme immobilization application, it promises exciting advantages for improving enzyme activity, stability, and engineering performances in bioprocessing and pharmaceutical industry applications.^12^, ^43^ Although these technologies and their potential are not fully exploited yet, they are gradually affecting many areas, especially the research, development, and production of homochiral drugs. The continuous advances in the areas of bioinformatics, recombinant DNA technologies, protein and strain engineering together with the interconnections between these areas will open the door to a wider use of biotechnological‐based industrial processes.
